# Combinations of registered drugs reduce treatment times required to deplete *Wolbachia* in the *Litomosoides sigmodontis* mouse model

**DOI:** 10.1371/journal.pntd.0006116

**Published:** 2018-01-04

**Authors:** Sabine Specht, Kenneth M. Pfarr, Sandra Arriens, Marc P. Hübner, Ute Klarmann-Schulz, Marianne Koschel, Sonja Sternberg, Coralie Martin, Louise Ford, Mark J. Taylor, Achim Hoerauf

**Affiliations:** 1 Institute for Medical Microbiology, Immunology and Parasitology, University Hospital Bonn, Bonn, Germany; 2 Institute for Laboratory Animal Science, Vetsuisse Faculty, University of Zurich, Switzerland; 3 German Centre for Infection Research (DZIF), partner site Bonn-Cologne, Bonn, Germany; 4 UMR 7245 MCAM MNHN CNRS, Museum National d`Histoire Naturelle, Paris, France; 5 Department of Parasitology, Liverpool School of Tropical Medicine, Liverpool, United Kingdom; University of California San Diego School of Medicine, UNITED STATES

## Abstract

Filarial parasites can be targeted by antibiotic treatment due to their unique endosymbiotic relationship with *Wolbachia* bacteria. This finding has led to successful treatment strategies in both, human onchocerciasis and lymphatic filariasis. A 4–6 week treatment course using doxycycline results in long-term sterility and safe macrofilaricidal activity in humans. However, current treatment times and doxycycline contraindications in children and pregnant women preclude widespread administration of doxycycline in public health control programs; therefore, the search for shorter anti-wolbachial regimens is a focus of ongoing research. We have established an *in vivo* model for compound screening, using mice infected with *Litomosoides sigmodontis*. We could show that gold standard doxycycline treatment did not only deplete *Wolbachia*, it also resulted in a larval arrest. In this model, combinations of registered antibiotics were tested for their anti-wolbachial activity. Administration of rifamycins in combination with doxycycline for 7 days successfully depleted *Wolbachia* by > 2 log (>99% reduction) and thus resulted in a significant reduction of the treatment duration. Using a triple combination of a tetracycline (doxycycline or minocycline), a rifamycin and a fluoroquinolone (moxifloxacin) led to an even greater shortening of the treatment time. Testing all double combinations that could be derived from the triple combinations revealed that the combination of rifapentine (15mg/kg) and moxifloxacin (2 x 200mg/kg) showed the strongest reduction of treatment time in intraperitoneal and also oral administration routes. The rifapentine plus moxifloxacin combination was equivalent to the triple combination with additional doxycycline (>99% *Wolbachia* reduction). These investigations suggest that it is possible to shorten anti-wolbachial treatment times with combination treatments in order to achieve the target product profile (TPP) requirements for macrofilaricidal drugs of no more than 7–10 days of treatment.

## Author summary

Over the past years, more attention has been brought to neglected tropical diseases including lymphatic filariasis and onchocerciasis. The latter are caused by helminthic parasites and lead to chronic and debilitating symptoms and present a major health burden that also affects the economy of endemic countries. It has been suggested that disease elimination may be possible but an accelerated implementation of proven and cost-effective interventions are needed if the targets for elimination are to be achieved. Recently, an indirect mode of action has been identified, targeting bacterial *Wolbachia* endosymbionts within the filariae, which also kills the adult parasites, an advantage over the drug currently used for mass drug administration, i.e. ivermectin. Doxycycline has been successfully used in clinical trials, however due to its long regimen as well as restrictions of use in children and pregnant women new drugs or drug combinations are required that overcome these obstacles. Here, we present the filarial parasite *Litomosoides sigmodontis* as suitable model for the preclinical testing of anti-wolbachial drugs against filariae and show that combinations of already registered drugs with anti-wolbachial efficacy are able to reduce the treatment time dramatically.

## Introduction

More than 200 million humans are parasitized by filarial nematodes, causing the neglected tropical diseases: lymphatic filariasis, loiasis and onchocerciasis. The lymphatic, ocular and dermatological damages have severe economic and social consequences including poor school performance, low productivity, low income, higher health related costs among infected adults, and a reduced life span [1]. The achievements of onchocerciasis and lymphatic filariasis (LF) mass drug administration (MDA) programmes have considerably reduced transmission and led to the formulation of the goal to eliminate these diseases [2]. However, research is still needed to develop safe and easy to administer macrofilaricides (drugs that kill the adult filarial parasites) to reduce the treatment time needed for MDA programmes and to be used in “problem areas”, e.g. areas in which suboptimal responses and potential resistance to ivermectin (IVM) have emerged or *Loa loa* is co-endemic. In the latter, MDA programmes have not been implemented due to the risk of severe adverse events in co-infected patients due to rapid killing of *L*. *loa* microfilariae (MF) by IVM [3]. The development and implementation of macrofilaricidal drugs will increase cost effectiveness by avoiding unnecessary IVM treatments, particularly in “end-game” scenarios or when shifting from MDA to “test & treat” strategies [4]. Furthermore, implementation of macrofilaricidal drugs will reduce the program times required to eliminate onchocerciasis and LF, which is the aim of the Millennium Development Goals.

Due to their mutualistic association with filarial worms, *Wolbachia* have been discovered as new target for chemotherapy in filariasis [5, 6]. Treatment of filarial infected animals with tetracycline resulted in the elimination of *Wolbachia* from filarial tissues in *Litomosoides sigmodontis*, *Brugia malayi*, *B*. *pahangi*, *Onchocerca ochengi*, *O*. *lienalis*, *O*. *gutturosa* and *Dirofilaria immitis*, prevented parasite establishment and filarial growth and rendered adult worms sterile [7–12]. *Wolbachia*-negative filarial species such as *Acanthocheilonema viteae* and *Loa loa* are not influenced by antibiotic treatment, suggesting that filarial viability and fertility is not affected directly [9, 13]. We have established the first safe macrofilaricide in humans [14–16], doxycycline (DOX), which targets essential *Wolbachia* endosymbionts, and has proven efficacy in both, lymphatic filariasis and onchocerciasis. An advantage of this mode of action is the slow onset of anti-parasitic activity, thereby avoiding adverse reactions caused by rapid micro- and/or macrofilaricidal activity [17]. However, treatment duration lasts 4–6 weeks and is not an option for children and pregnant or lactating women. The anti-*Wolbachia* (A-WOL) consortium aims to identify novel anti-*Wolbachia* drugs, compounds or combinations that are suitable for use in humans [4]. *In vitro* experiments using a *Wolbachia*-containing insect cell culture [18] have provided a valuable tool for high throughput screening for anti-wolbachial compounds [19]. As a second step, candidates successful in *in vitro Wolbachia*-depletion assays have to be tested for their *in vivo* efficacy, their bioavailability within the mammalian host as well as within the parasite. Choosing the right parasite model and the optimal protocol / time-line is a critical issue to balance high-throughput screening potential and ability to translate results for efficacy or shortening of treatment time against the human parasite. Despite general similarities, each of the existing filarial models has its strengths and limitations [20] and the discovery of macrofilaricidal drugs, i.e. killing directly the adult parasite, has been a critical issue due to varying parasite sensitivity to the drug or bioavailability of the drug within the different hosts used.

Since in anti-wolbachial therapy, the mode of action has been elucidated and it is known that the depletion of *Wolbachia* eventually leads to parasite death [15], the first step of proving a drug´s *in vivo* activity is to show that indeed *Wolbachia* are depleted with a given regimen. Here we present natural infection of BALB/c mice with the filarial nematode *L*. *sigmodontis* ([21], formerly known as *Litomosoides carinii* and later re-named by Bain *et al*. [22]) as a rodent model for an *in vivo* screening of anti-wolbachial compounds. The choice of this parasite / host combination was driven by the following advantages: i) immunocompetent mice are susceptible to *L*. *sigmodontis*, ii) parasites develop much faster compared to *O*. *ochengi* in cattle, iii) the model allows a quick optical screen on day 35 for worms with a stunted growth as a correlate to anti-wolbachial activity, iv) if a macrofilaricidal activity of hits in an already patent infection shall be analysed, the same parasite species can be used in larger rodents, such as Mongolian gerbils, which allow longer follow up times suitable for the slow macrofilaricidal action of anti-wolbachials. In the present study, we use the larval *L*. *sigmodontis* model for anti-wolbachial screening and describe the effects of doxycycline at doses that provide sufficient and suboptimal drug exposure *in vivo*. This enabled us to compare the treatment time of compounds to deplete *Wolbachia* directly against DOX and revealed that minocycline has a superior efficacy. In addition, rifamycins were tested and rifapentine (RPT) was proven to be more efficacious compared to rifampicin. Finally, combination therapies were investigated and the combination of RPT and moxifloxacin (MOX) showed the strongest reduction of treatment time using both intraperitoneal (ip) and oral administration routes and allowed a strongly reduced treatment time from 14 days to only 2 and 4 days, respectively. These investigations suggest that it may be possible to shorten anti-wolbachial treatment times to match the current target product profile (TPP) for macrofilaricidal drugs [4].

## Materials and methods

### Ethics statement

Animal housing conditions and the procedures used in this work complied with to the European Union animal welfare Directive 2010/63 EU. All protocols were approved by the Landesamt für Natur, Umwelt und Verbraucherschutz, Cologne, Germany (AZ 8.87–50.10.35.08.024, AZ 84–02.04.2012.A140).

### Study design

Experiments were designed to compare *Wolbachia* reduction in *L*. *sigmodontis* worms between different treatment groups, a “doxycycline gold standard" (50 mg/kg/day administered ip for 14 days) and the vehicle control, in a randomized design with multiple arms and a shared vehicle control. The primary endpoint was the *Wolbachia* single gene FtsZ copy number compared to the vehicle control. As secondary objective FtsZ levels as well as the FtsZ/worm actin ratio were further compared to the gold standard treatment [23]. Sample size calculation was based on the mouse as experimental unit using the mean and SD of the FtsZ copy numbers of 10 worms per experimental unit. At an alpha-level of 0.05 and a power of 0.95 with an effect size of d = 8.18, the estimated groups size was 2 due to the strong *Wolbachia* reduction efficacy of the gold standard. This calculation was based on a preliminary experiment with five worms per animal and three animals per group. To compensate for possible variation in infections, at least three animals were used in the experimental and control groups. All possible measures were taken to minimize nuisance or the effects of subjective bias when allocating animals to treatment (computer-generated random sequence numbers). Using 4–6 week old female BALB/c mice from one supplier, the bias of the experimental unit was minimized. Furthermore, all animals in one experiment were infected at the same time with the same batch of parasites in order to reduce the variation of infection to the minimum. After the infection, animals were randomly assigned to the treatment groups. Results were assessed in a blinded manner, as the person involved in animal treatment and the person analysing the FtsZ levels were not aware of the treatment groups.

### Animal and parasite maintenance

Female BALB/c mice were purchased from Janvier Labs, Saint-Berthevin, France, at the age of 4–6 weeks and were allowed to adjust for 7 days prior to infection. Animals were housed in the animal facilities (conventional, OHB (optimized hygiene barrier)) of the University Hospital of Bonn in individually ventilated cages with access to food and water ad libitum and enrichment. The *L*. *sigmodontis* life cycle was maintained at the Institute for Medical Microbiology, Immunology and Parasitology as described earlier [24]. *L*. *sigmodontis* lives naturally in the cotton rat (*Sigmodon hispidus*). During the blood meal on an infected cotton rat the arthropod intermediate host (tropical rat mite *Ornithonyssus bacoti*) ingests the microfilariae (MF) which moult twice and develop to the infective stage 3 larvae (L3) within 10 days. In a following blood meal the mites transmit the L3 to cotton rats. Similarly, in the case of murine infection the L3 containing mites are allowed to take blood from mice and thereby transmit the L3. In the rodent host the L3 migrate to the thoracic cavity and reach sexual maturity within 25–33 days.

### Infection and treatment

Six to eight week old female BALB/c mice were infected with *L*. *sigmodontis* by natural infection as described above. Beginning one day after infection, the mice were given ip injections of 10% DMSO (vehicle control), drugs as indicated in the text and figures, or were left untreated. If not indicated otherwise, all substances were obtained from Sigma Aldrich with a purity of >90% and diluted in PBS, 10% DMSO. Gold standard (DOX ip, 50 mg/kg/day,) and vehicle control were given for 14 days. So adverse events have been observed during treatment. Thirty-five days post infection, the animals were euthanized using isoflorane and worms were recovered from the thoracic cavity by PBS lavage. The female worms were sorted, their lengths measured and individually frozen for DNA extraction. At least 3 mice and 5–10 *L*. *sigmodontis* females per mouse were used for each treatment group.

### DNA extraction and PCR

Genomic DNA was extracted from individual worms using the QIAamp DNA mini kit (Qiagen, Hilden, Germany). The Qiagen protocol was used with the following changes: the worms were incubated with proteinase K overnight at 56°C; and Wizard SV96 DNA binding plates (Promega) and vacuum manifold instead of DNA columns were used to bind, wash, and elute the DNA in 50 μL of 10-mM Tris, 0.5-mM EDTA, pH 9. Elution plates were sealed and stored at −20°C.

Reduction/depletion of *Wolbachia* was monitored by qPCR using Rotorgene (Qiagen) and primers for *Wolbachia* FtsZ (GenBank Accession No.: AJ010271), a single copy number gene. Filarial actin (GenBank Accession No.: GU971367) was determined to normalize worms of a different size. The genes were quantified from the purified DNA by real-time duplex PCR (qPCR) using the Qiagen’s QuantiTect Multiplex NoROX Kit with the following conditions: 10x HotStar Taq Polymerase buffer (Qiagen), 200 μM dNTP, Primers: LsFtsZ forward primer (5´-cgatgagattatggaacatataa-3´), LsFtsZ reverse primer (5´-ttgcaattactggtgctgc-3´), LsActin forward primer (5´-atccaagctgtcctgtctct-3`), LsActin reverse primer (5´-tgagaattgatttgagctaatg-3´), LsFtsZ taqman probe (5’6-FAM cagggatgggtggtggtactggaa 3’ TAMRA), LsActin taqman probe (5’HEX actaccggtattgtgctcgatt 3’TAMRA), 2.5 units HotStar Taq, and 2 μl DNA in a 20 μl reaction. Final primer concentrations were 500 nM for LsFtsZ and 400 nM for LsActin, final taqman probe concentrations were 25 nM for LsFtsZ and 50 nM for LsActin and 6 mM for the MgCl_2_ concentration. Genes were amplified in a Rotorgene 3000 (Qiagen) using the following conditions: 1X 15 min at 95°C, 45 cycles of 95°C for 15 sec, 58°C for 30 sec. Fluorescence was acquired on the FAM and JOE channel. Copy numbers for each gene were calculated using a modification of the comparative quantification formula as described in [23]. FtsZ and Actin copy numbers are given per 50 μl single worm DNA extract, of which 2 μl were used for each PCR run.

### Statistics

Data were distributed in a non-parametric fashion, median and interquartile ranges are presented. For comparing the level of *Wolbachia* depletion in worms, the Mann-Whitney-U test was used to calculate statistical differences either against the vehicle treated or gold standard groups. All *p*-values are given in S1 Table. Analyses between different experimental groups are given in the figures, when additionally performed. *P*-values <0.05 were considered to represent significant differences. All statistics were calculated using Graph- Pad Prism version 5.02 for Macintosh.

## Results

### *Wolbachia* depletion by doxycycline in the *L*. *sigmodontis* rodent model

**Fig 1:**
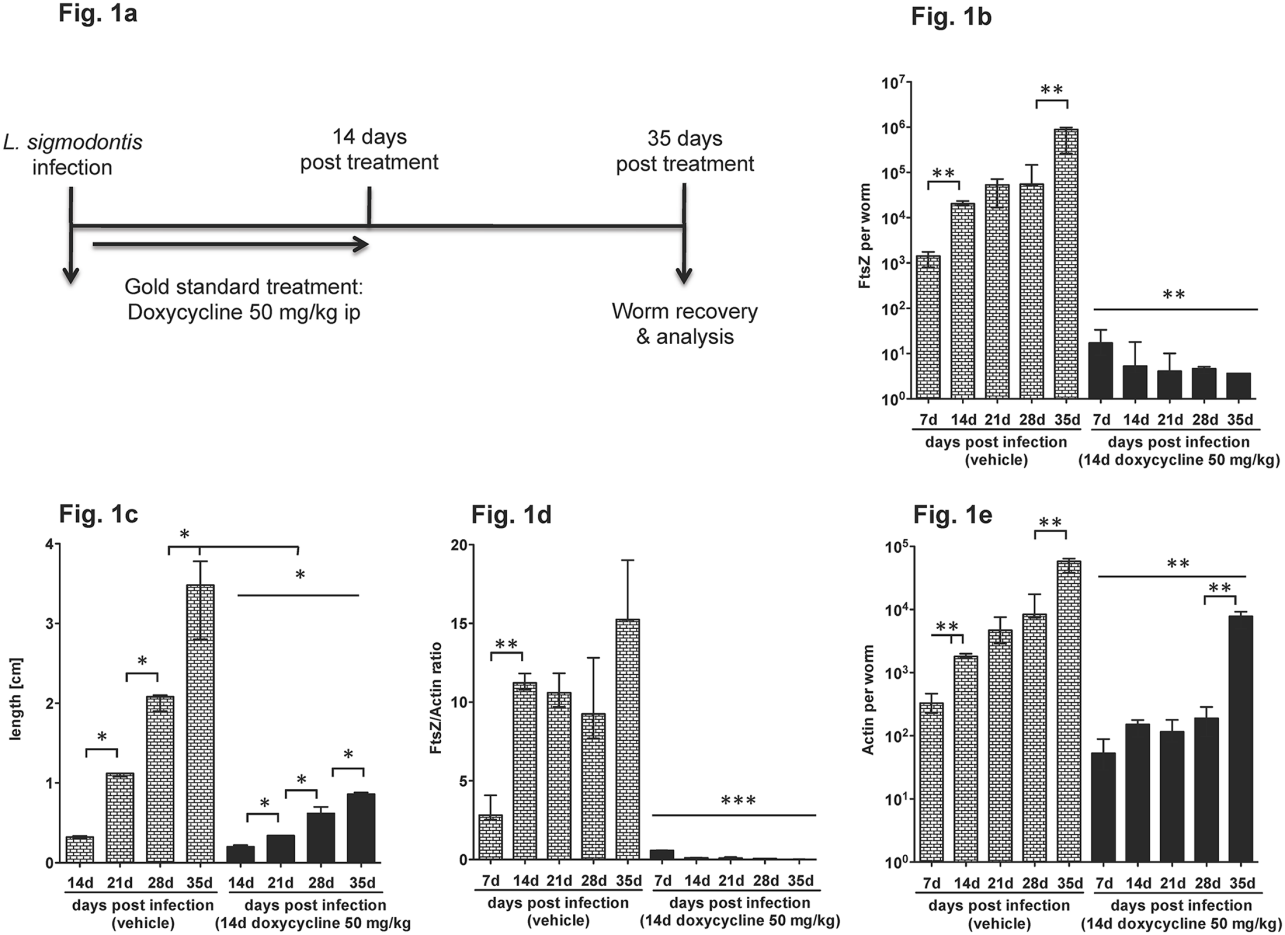
*Wolbachia* depletion and reduction in worm length after doxycycline treatment. (**a**) Experimental set up for investigation of anti-*Wolbachia* compounds. BALB/c mice are naturally infected with the filarial parasite *Litomosoides sigmodontis* and treatment starts the following day. As positive control 50mg/kg/d doxycycline (DOX) and vehicle as negative control were administered for 14 days intraperitoneally. At 7–35 days post infection worms were recovered from the pleural cavity, staged, measured for length and DNA was extracted and analysed for *Wolbachia* FtsZ and *L*. *sigmodontis* actin from single female worms. FtsZ copy numbers (**b**), female worm length (**c**), filarial actin copy numbers (**d**) and the FtsZ/actin ratio (**e**) are given from worms recovered from mice receiving DOX intraperitoneally at 50 mg/kg/d for 14 days. Worms were recovered at 7, 14, 21, and 35 days post infection. Significances were tested with Mann-Whitney-U test. *p<0.05; **p<0.01; ***p<0.001.

Experiments were conducted in BALB/c mice, infected naturally with the rodent filarial parasite *L*. *sigmodontis* via the bite of tropical rat mites (*Ornithonyssus bacoti*). Starting the following day, animals were subjected to a daily ip injection of 50 mg/kg (mg/kg) of DOX for 14 days (Fig 1a) and filariae were recovered and analysed after 7 (only 7 days of dosing), 14, 21, 28 and 35 days post infection (dpi) for their *Wolbachia* content. Fig 1b shows that in DOX treated female worms *Wolbachia* FtsZ copy numbers were decreased from 7 dpi throughout day 35 pi (Fig 1b). In untreated animals *Wolbachia* copy numbers increased between day 7 to 14 and 28 to 35 pi, indicating a rapid multiplication of the bacterial endosymbionts during larval development. The increase of bacteria in untreated controls coincides with their moult from L3 into L4 and L4 into young adults. We further investigated the size of the female worms starting at day 14 pi and found a growth retardation after DOX treatment with parasites that did not develop beyond the size of untreated filariae at day 21 pi (Fig 1c). Accordingly, filarial actin copy numbers increased in vehicle treated controls from 7 to 35 dpi (Fig 1d). As a result, the ratio of *Wolbachia* FtsZ over filarial actin showed an increase between days 7 to 14 and 28 to 35 pi in vehicle treated animals, which was reduced by 96.4 to 99.9% by DOX treatment (Fig 1e).

With this model as a screening tool for anti-wolbachial compounds, it is possible to detect effective compounds by day 14 pi. In the following experiments we chose to use day 35 pi for *Wolbachia* measurements as this time point has the advantage of a second parameter, i.e. growth inhibition of the worms for a rapid evaluation of compounds.

### *Wolbachia* depletion by doxycycline leads to growth inhibition of *L*. *sigmodontis*

**Fig 2:**
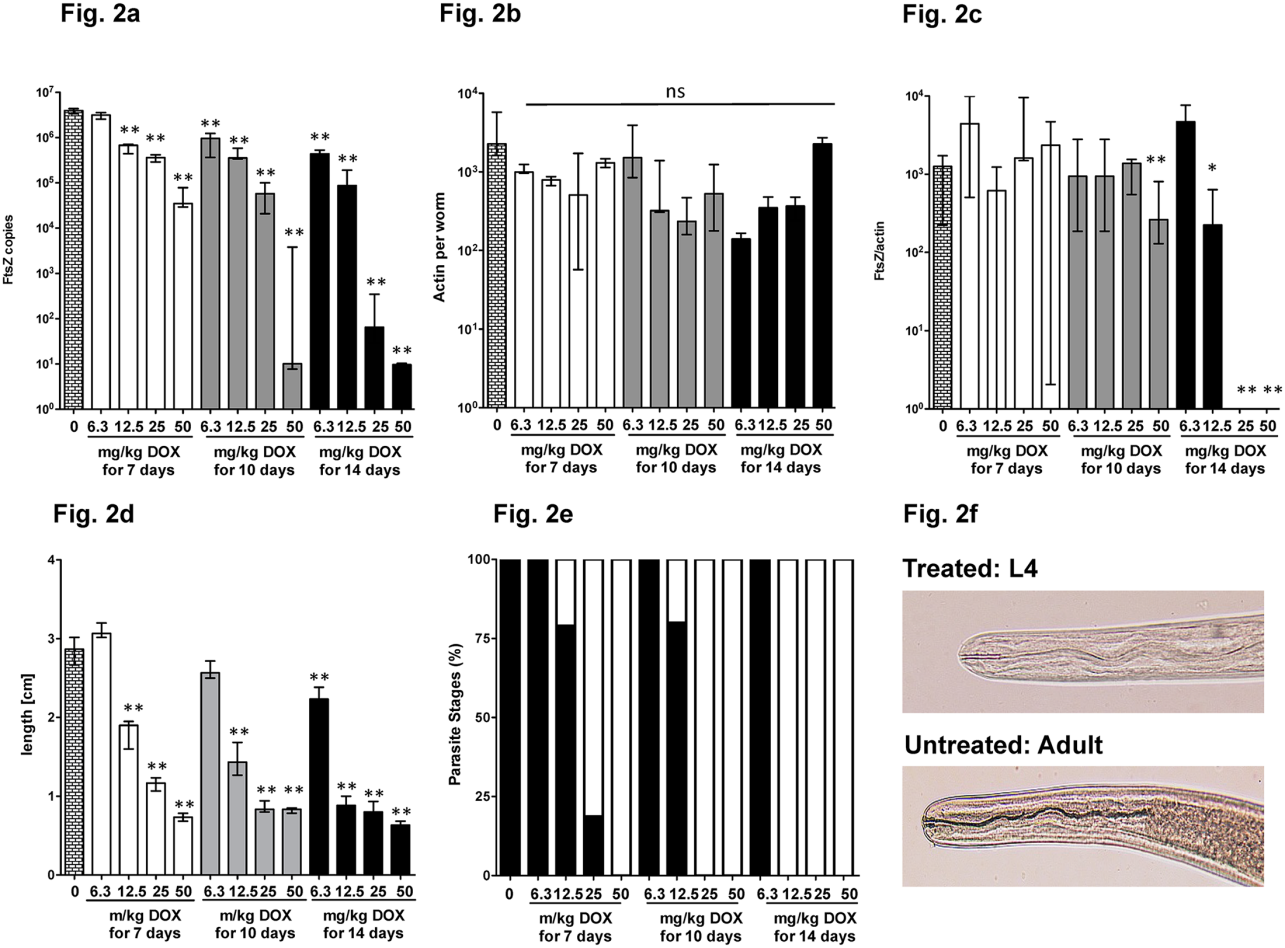
Titration of intraperitoneally administered doxycycline for *Wolbachia* depletion. Doxycycline (DOX) was administered intraperitoneally at the concentrations indicated for either 7, 10 or 14 days. Worms were recovered at day 35 post infection. (**a**) FtsZ and (**b**) Actin copy numbers as well as (**c**) FtsZ/Actin ratio are given per single female worm. (**d**) Female worm length and (**e**) parasite stage (adult—black bars, L4—white bars). (**f**) Representative pictures demonstrating the buccal capsule as differentiator for L4 larvae and adult worms. Significances were tested with Mann-Whitney-U test. *p<0.05; **p<0.01; ***p<0.001 compared to vehicle treated. Exact p-values are given in S1 Table.

Next, we investigated optimal as well as suboptimal DOX treatments for *Wolbachia* depletion in order to set up standards for identification of compounds that are more potent than DOX. To this, we subjected infected animals to a 7, 10 and 14 day treatment of daily ip DOX doses ranging from 6.3 to 50 mg/kg and performed the analysis of female worms at 35dpi. *Wolbachia* levels were reduced in a dose and treatment time dependent manner (Fig 2a), whereas parasite actin DNA levels were not significantly affected by the treatment (Fig 2b). The ratio of *Wolbachia* FtsZ and filarial actin DNA was also calculated and is presented in Fig 2c. Examination of the size and developmental stage of the worms revealed a dose and treatment time dependent inhibition of filarial growth (Fig 2d) in accordance to the loss of *Wolbachia*. In addition, parasites that were recovered from animals with higher DOX dose regimens and longer treatment times remained in the L4 larval stage as determined based on the length of the buccal capsule (white bars, Fig 2e and 2f), suggesting that growth and development is impaired by depletion of *Wolbachia*. Interestingly, light microscopy analysis indicated no morphological damages, e.g. with regard to the integrity of male spiculae (personal communication Dr. Coralie Martin). Growth inhibition occurred already at lower drug doses that did not achieve the threshold of >99% *Wolbachia* reduction, indicating that the treatment affected the functional activity of the bacteria and growth inhibition could be achieved with suboptimal *Wolbachia* reduction. In addition, we analysed the FtsZ and actin signal in the vehicle and gold standard groups (14 days ip 50 mg/kg/d DOX) from all our experiments (n = 23). After DOX, FtsZ levels were reduced on average by 5 log drops, whereas actin levels varied by a maximum of one log. We found that for FtsZ the median was 36 (±8.15x10^4^) in the worms recovered after DOX and 6.6x10^6^ (±1.35x10^6^) in the vehicle group and for the nematode actin 3,4x10^4^ (±7.1 x10^4^) in the DOX and 5.5 x10^5^ (±1.1 x10^6^) in the control group.

Taken together these data show that 50 mg/kg DOX ip for 14 days is suitable to deplete *Wolbachia* by >99%. For the detection of compounds that are more potent than DOX, a suboptimal regimen, e.g. 25 mg/kg DOX for 10d, is recommended. This dose results in a small but significant reduction of FtsZ copy numbers and more prominently a clear inhibition of growth, suggesting that functional impairment of *Wolbachia* (e.g.production of molecules that enable parasite development) precedes their disappearance from the parasite tissue. Having set the time point of analysis at day 35 pi, it is possible to identify active compounds quickly by reduction in worm length, even if FtsZ is not or only marginally reduced. Thereby potential drug candidates can be identified with higher potency and treatment regimens can be optimized.

**Fig 3:**
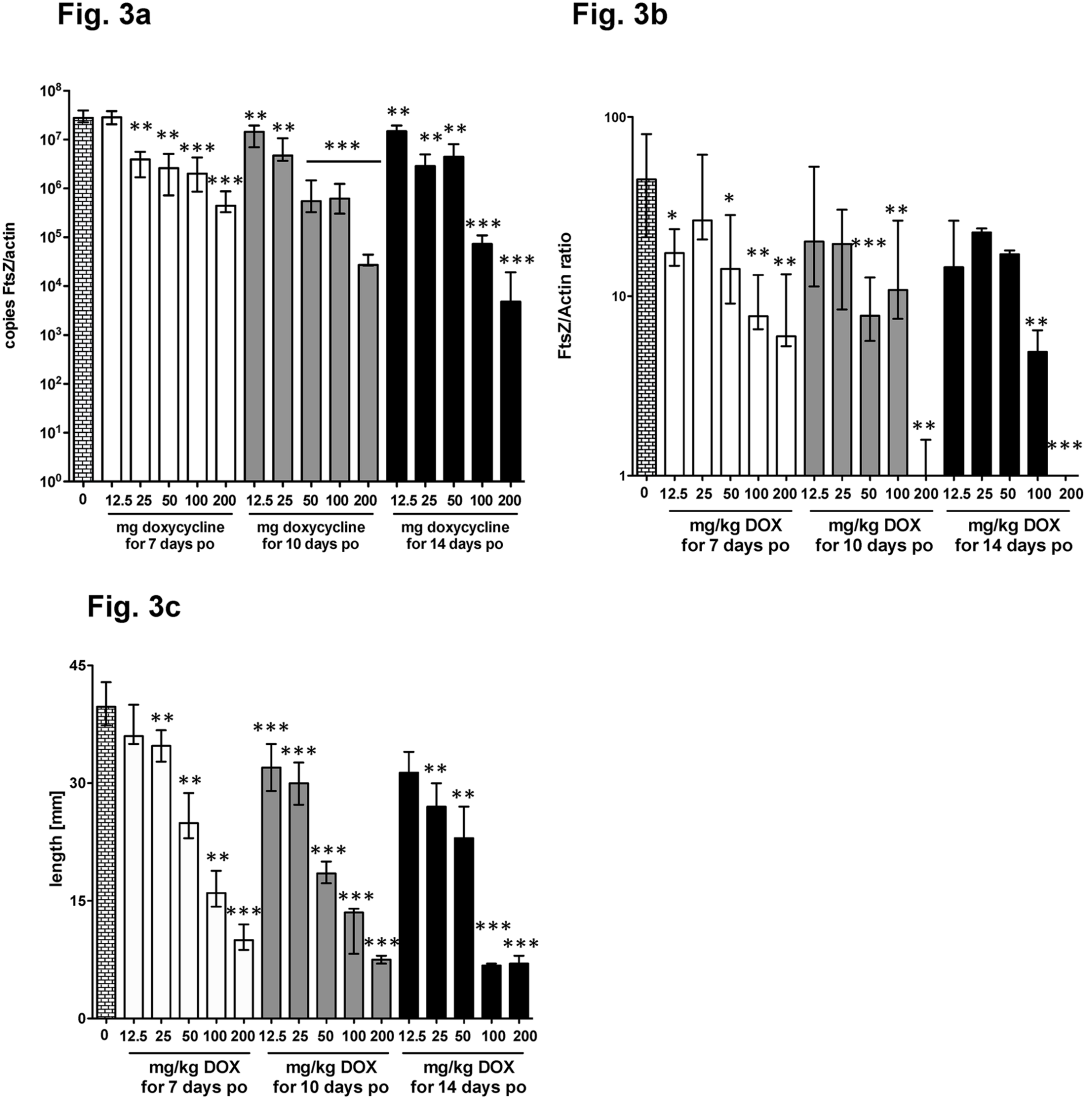
Titration of orally administered doxycycline for *Wolbachia* depletion. Doxycycline (DOX) was administered daily by oral gavage at the concentrations indicated for either 7, 10 or 14 days. Worms were recovered at day 35 post infection. (**a**) FtsZ copy numbers and (**b**) FtsZ/Actin ratio per single female worm. The female worm length is shown in **(c)**. Significances were tested with Mann-Whitney-U test. *p<0.05; **p<0.01; ***p<0.001. Exact p-values are given in S1 Table.

According to the current target product profiles, new macrofilaricidal drugs should have good oral bioavailability. Therefore we performed a dose titration with oral DOX. A dose response in FtsZ reduction was observed when DOX was administered orally (Fig 3a). Also the FtsZ/actin ratio (Fig 3b) and the length reduction (Fig 3c) showed a dose response. Despite the fact that treatment duration may not have been long enough for complete absence of the *Wolbachia* signal, growth inhibition occurred at 200 mg/kg for 7/10/14 days and 100 mg/kg for 14d and indicates effective inactivation of *Wolbachia*.

### Validation of the model with known antibiotics

**Fig 4:**
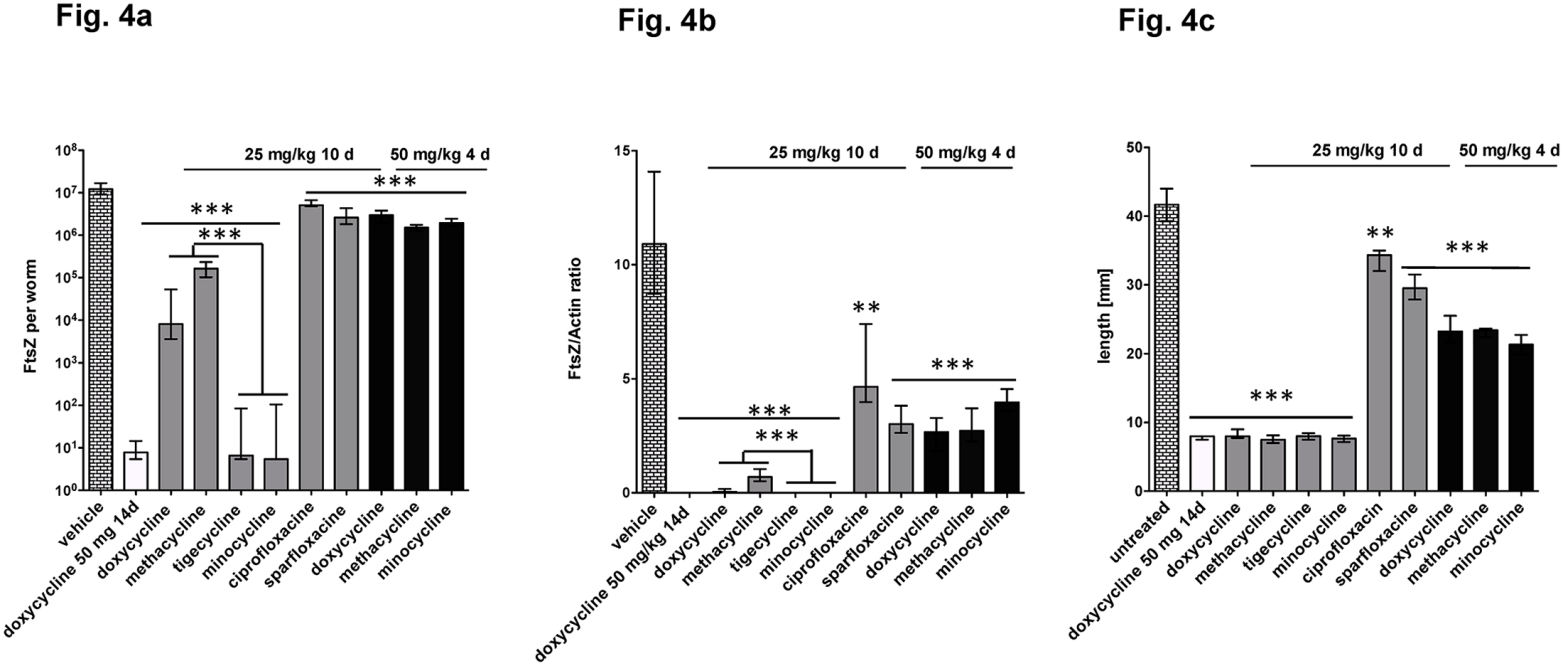
Comparison of different antibiotics for *Wolbachia* depletion. Antibiotics were administered intraperitoneally at 25mg/kg/d for 10 days or 50mg/kg/d for 4 days. Worms were recovered at day 35 post infection. (**a**) FtsZ copy numbers and (**b**) FtsZ/Actin ratio are given per single female worm. The female worm length is shown in (**c**). Significances were tested with Mann-Whitney-U test. *p<0.05; **p<0.01; ***p<0.001. Exact p-values are given in S1 Table.

We tested the ability of the *L*. *sigmodontis* model to identify compounds with improved activity compared to DOX, using a series of registered antibiotics with known or unknown anti-wolbachial activity. Among these were tetracycline derivatives (tigecycline, minocycline, methacycline) and gyrase inhibitors (sparfloxacin, ciprofloxacin). Animals were subjected to a 10 day (all compounds at 25 mg/kg per day) or 4 day ip treatment course (tetracyclines at 50 mg/kg per day). All tetracyclines administered for 10 days inhibited filarial growth, whereas both gyrase inhibitiors were less effective (Fig 4). Sparfloxacine was reported to be effective at higher doses (160 mg/kg, 14 days) [19].

FtsZ copy numbers further show that tigecycline and minocycline were more potent than DOX (Fig 4a and 4b). Shortened treatment for 4 days with the tetracyclines methacycline and minocycline resulted in a less pronounced *Wolbachia* decline (83.8 and 88.0% reduction of FtsZ; Fig 4a) and partial growth inhibition (Fig 4c).

### Rifamycins are highly active against *Wolbachia*

**Fig 5:**
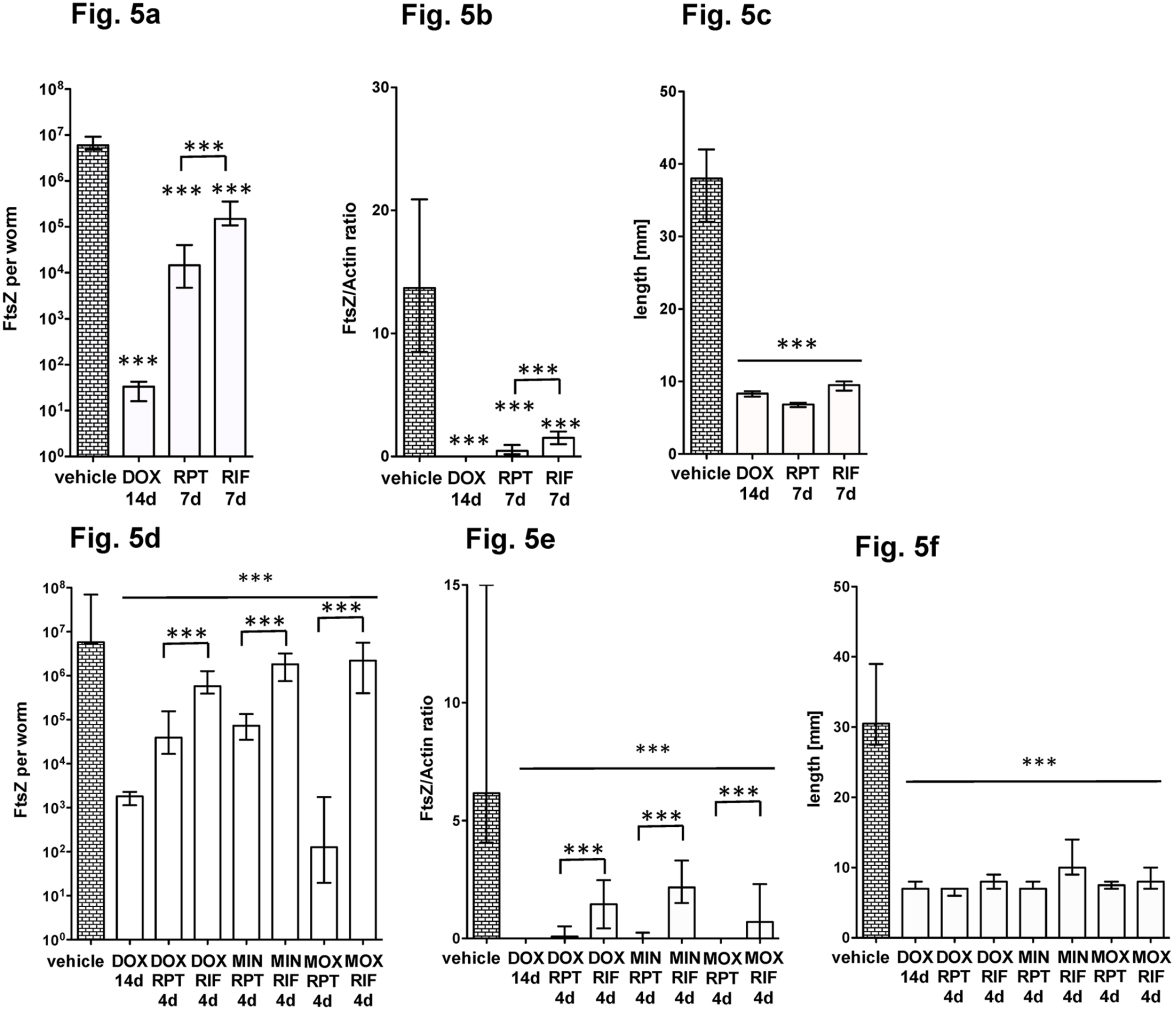
Comparison of antibiotic/rifamycin double-combinations for *Wolbachia* depletion. Rifamycins and antibiotics were administered intraperitoneally at 50 mg/kg/d and moxifloxacin (MOX) at 200 mg/kg/bid. Worms were recovered at day 35 post infection. (**a, d**) FtsZ copy numbers, (**b, e**) FtsZ/Actin ratios and (**c, f**) female worm length of mice treated with rifapentine (RPT), rifampicin (RIF) or doxycycline (DOX) alone (**a-c**), or in combinations of DOX, minocycline (MIN) and MOX with RPT or RIF (**d-f**). Significances were tested with Mann-Whitney-U test. *p<0.05; **p<0.01; ***p<0.001. Exact p-values are given in S1 Table.

We next investigated whether rifamycins are equivalent or faster acting than DOX and analysed rifapentine (RPT) and rifampicin (RIF). Fig 5a–5c show that at a dose of 50 mg/kg ip for 7 days, RPT showed a slight but significant higher activity against *Wolbachia* compared to RIF (Fig 5a and 5b). Administration of both rifamycins reduced female worm length (Fig 5c).

A combination of drugs can reduce the time of treatment regimens, such as the Denver regimen against TB (MOX in combination with RPT, [25]) and the efficacy of a tetracycline / rifampin combination has already been shown against *Onchocerca ochengi*, albeit with longer treatment times [26]. We investigated the treatment time of such combinations in this model. We also included minocycline, as it was more potent than DOX (Fig 4). As we expected a high efficacy against *Wolbachia*, we administered the combination therapy for four days ip. Fig 5d–5f show that similar to the single administration, RPT is more potent than RIF in double combinations. Furthermore, minocycline (MIN) has similar activity as DOX in combination with either RPT or RIF. We also tested whether the MOX/rifamycin combination, as used against tuberculosis, is effective in this model. Whereas MOX alone was only partially effective at 7 dpi, MOX/RPT treatment for only four days showed *Wolbachia* depletion equivalent to the DOX gold standard administered for 14d (Fig 5d and 5e).

### Triple combination of a tetracycline, rifapentine and moxifloxacin is highly effective against *Wolbachia* endosymbionts

**Fig 6:**
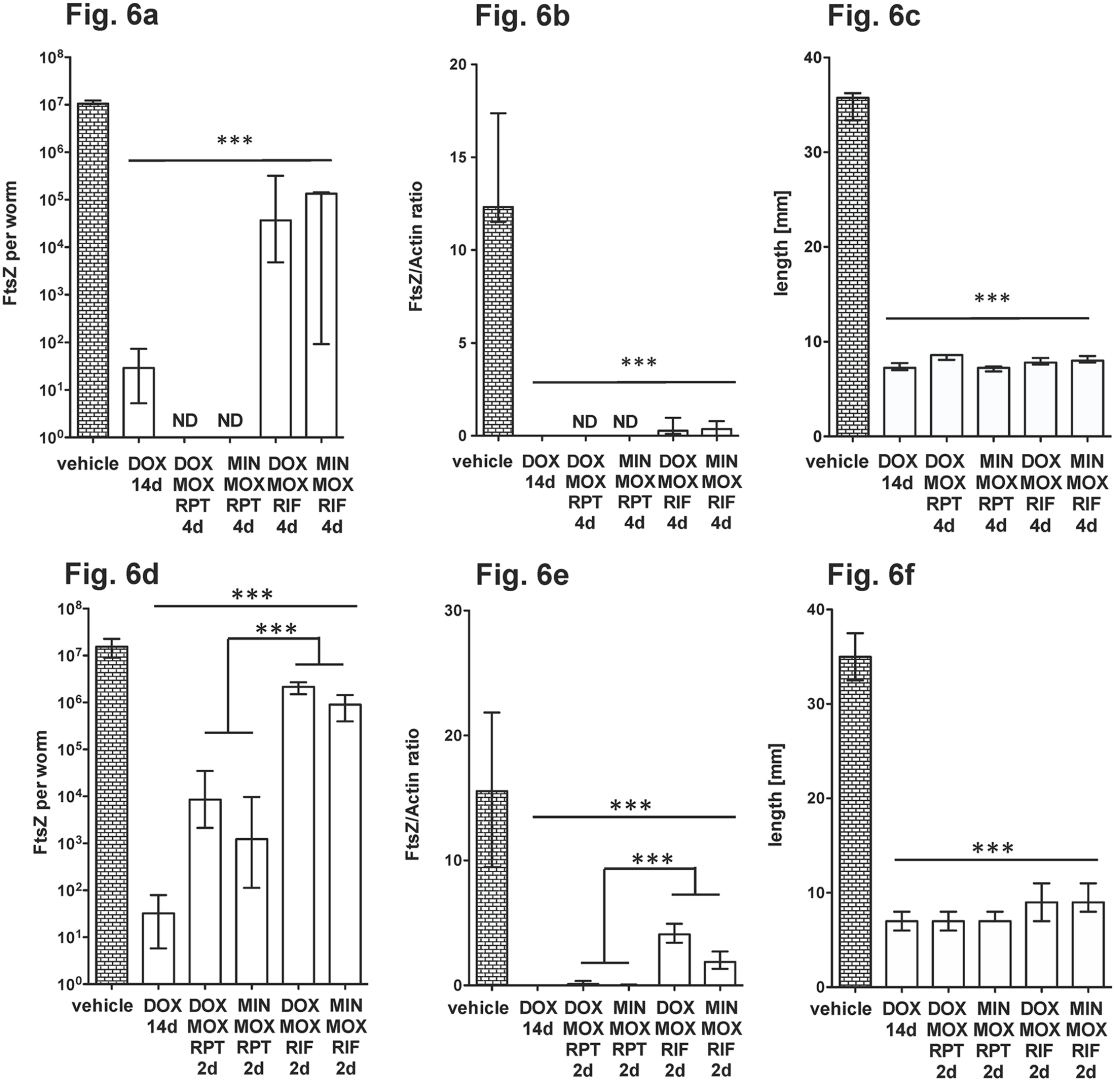
Comparison of antibiotic/rifamycin/moxifloxacin triple-combinations for *Wolbachia* depletion. Antibiotics and rifamycins were administered intraperitoneally at 50 mg/kg/d and moxifloxacin at 200 mg/kg/bid. Worms were recovered at day 35 post infection. (**a, d**) FtsZ copy numbers, (**b,e**) FtsZ/Actin ratio and female worm length (**c, f**) of mice treated for 4 (**a-c**) or 2 days (**d-f**). The female worm length is shown in (C, F). Significances were tested with Mann-Whitney-U test. *p<0.05; **p<0.01; ***p<0.001. Exact p-values are given in S1 Table.

**Fig 7:**
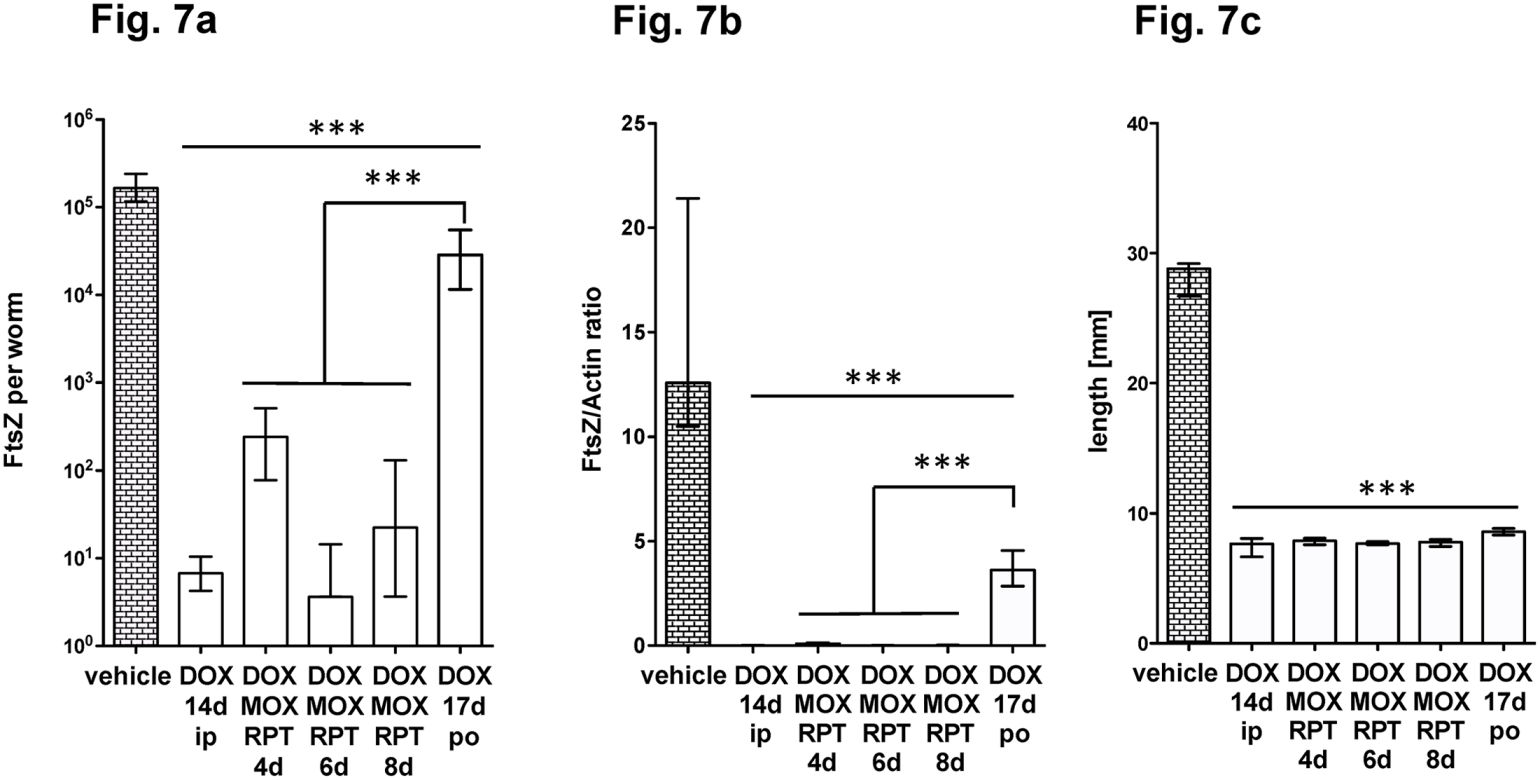
Oral administration of antibiotic/rifamycin/moxifloxacin triple-combinations for Wolbachia depletion. Antibiotics and rifamycins were administered ip at 50 mg/kg and moxifloxacin at 200 mg/kg/bid. Worms were recovered at day 35 post infection. Worms were recovered at day 35 post infection. FtsZ copy numbers (a) and the FtsZ/Actin ratio (b) are given per single female worm DNA extract. The female worm length is shown in (c). Significances were tested with MW-U test, a p-value of 0.05 was considered significant and exact p-values are given in S1 Table.

Since some of the double combinations showed efficacy when administered for 4 days, we investigated whether the addition of a triple combination of tetracycline, rifamycin and MOX further reduced the treatment time. We compared DOX (50 mg/kd/d), MIN (50 mg/kd/d), RPT (15 mg/kd/d), RIF (15 mg/kd/d) and MOX (200 mg/kd/bid) in a triple ip combination for 2 and 4 days. As seen before, all combinations were more efficacious if they contained RPT instead of RIF and all treatments tested inhibited filarial growth (Fig 6a–6f). Combining a tetracycline with RPT and MOX resulted in the greatest reduction of *Wolbachia*. Even a treatment time of 2 days depleted *Wolbachia* FtsZ by 99.9% when used in combination with RPT, but not with RIF. This finding indicates that anti-wolbachial drugs combinations reduce the treatment time from 14 days to 2 days in the *L*. *sigmodontis* larval model. We further tested the triple combination using the oral route and found that 4, 6 or 8 days of treatment reduced the number of bacteria significantly (>99.8%, Fig 7a and 7b) and impaired filarial growth (Fig 7c).

### A combination of MOX and RPT is effective against *Wolbachia*

**Fig 8:**
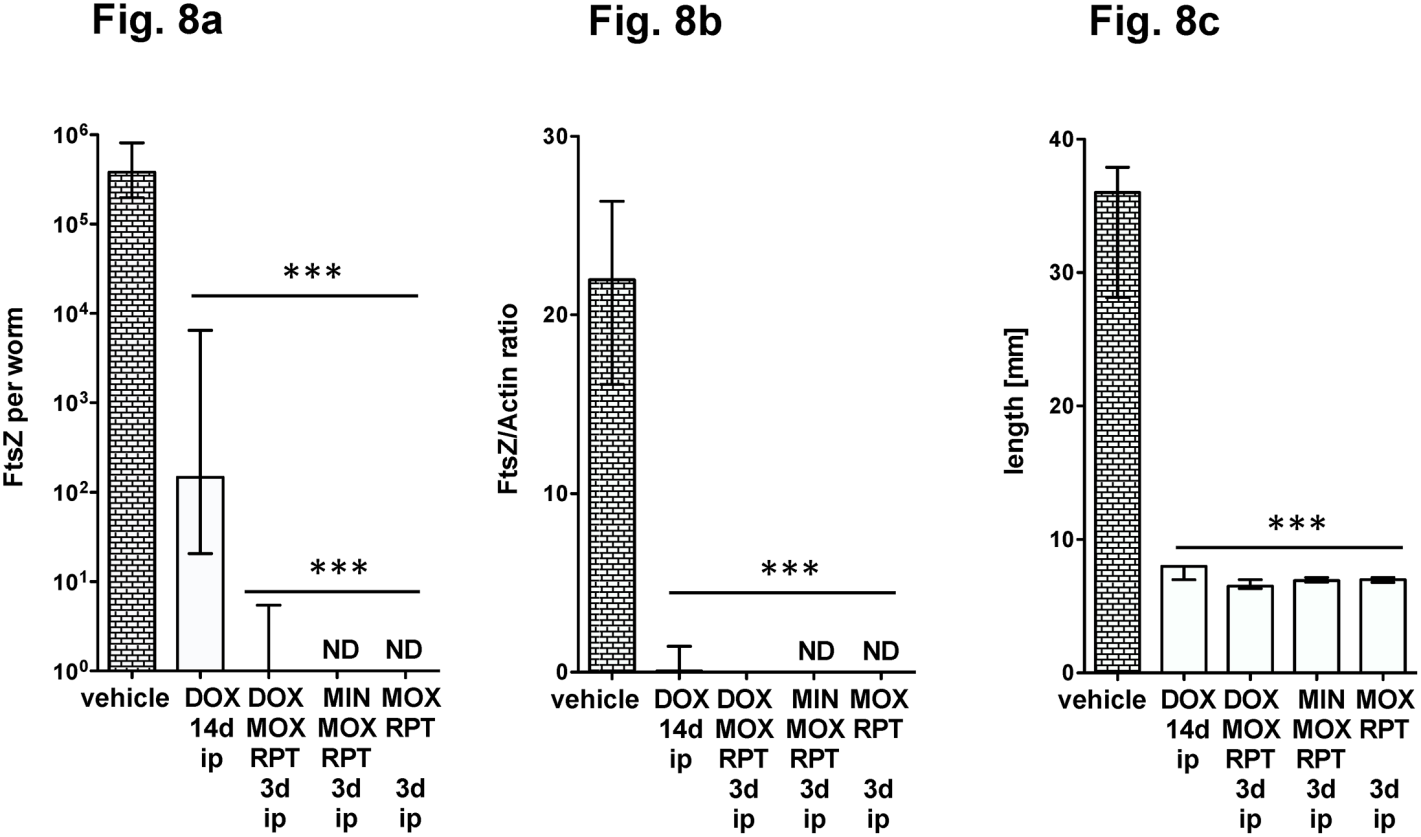
Comparison of antibiotic/rifamycin/moxifloxacin triple-combinations and RPT/MOX double-combination for *Wolbachia* depletion. Antibiotics were administered ip at 50 mg/kg, RPT at 15mg/kg and moxifloxacin at 200 mg/kg/bid. Worms were recovered at day 35 post infection. FtsZ copy numbers (a) and the FtsZ/Actin ratio (b) are given per single female worm DNA extract. The female worm length is shown in (c). Significances were tested with MW-U test, a p-value of 0.05 was considered significant and exact p-values are given in S1 Table.

**Fig 9:**
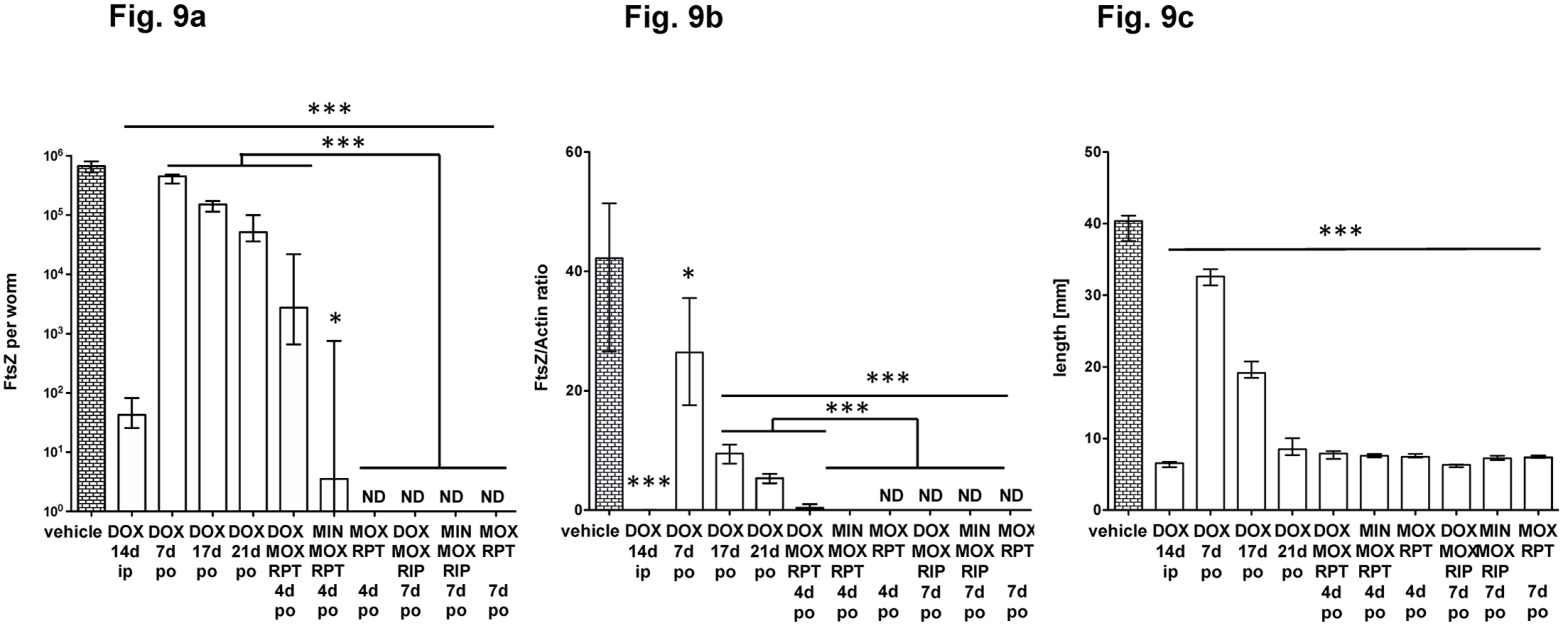
Comparison of antibiotic/rifamycin/moxifloxacin triple-combinations and RPT/MOX double-combination administered orally. Antibiotics were administered po at 50 mg/kg, RPT at 15mg/kg and moxifloxacin at 200 mg/kg/bid. Worms were recovered at day 35 post infection and DNA extracted. FtsZ copy numbers (a) and the FtsZ/Actin ratio (b) are given per single female worm DNA extract. The female worm length is shown in (c). Significances were tested with MW-U test, a p-value of 0.05 was considered significant and exact p-values are given in S1 Table.

In Fig 5d–5f we observed that also the double combination of ip RPT and MOX was effective in reducing *Wolbachia*. We compared this combination against a triple combination that included an additional tetracycline. Interestingly, when administered ip for 3 days at a dose of 15 mg/kg (RPT) and 200 mg/kg/bid (MOX), the double combination was equally effective to the triple combination including either 50 mg/kg DOX or MIN (Fig 8a–8c). Similarly, oral treatments for 4 or 7 days with the double combination of MOX and RPT was as effective in reducing *Wolbachia* as the triple combinations (Fig 9a–9c).

**Fig 10:**
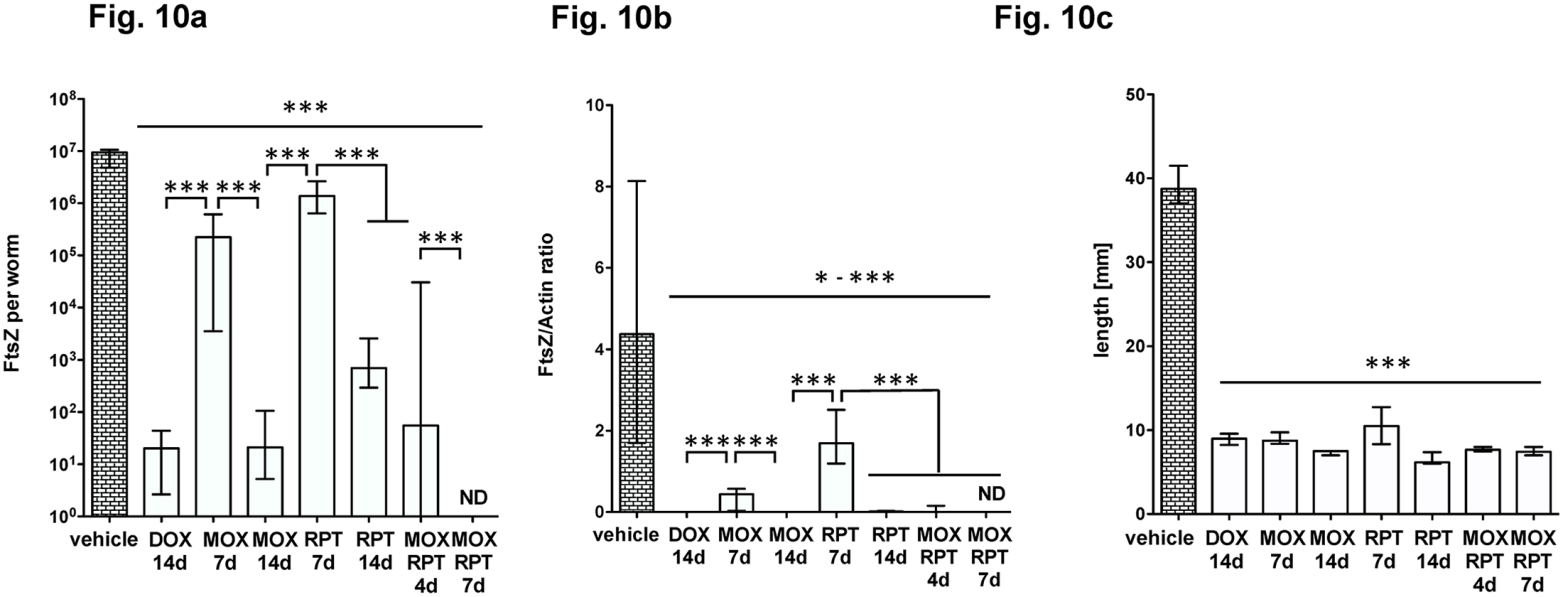
Lower dose MOX in a RPT/MOX double-combination is effective. Antibiotics were administered ip at 50 mg/kg, RPT at 15mg/kg and MOX 100 mg/kg ip/d. Worms were recovered at day 35 post infection. FtsZ copy numbers (a) and the FtsZ/Actin ratio (b) are given per single female worm DNA extract. The female worm length is shown in (c,). Significances were tested with MW-U test, a p-value of 0.05 was considered significant and exact p-values are given in S1 Table.

For the previous combinations a high MOX dose regimen was used and we investigated whether a lower MOX dose leads to the same results. Therefore we applied 15 mg/kg (RPT) and 100 mg/kg (MOX) and compared 7 and 14 days of treatment with the single components to 4 and 7 days of treatment with the combination. Fig 10a–10c shows that even at a lower MOX dose a significant *Wolbachia* reduction was achieved by the combination after 4 days of treatment (99.8%) and a reduction of 99.99% at 7 days of treatment. The single components MOX and RPT were both partially active with 7 days of treatment (96.8% and 83.6%) and highly active with 14 days of treatment (99.99% and 99.6%).

Taken together, a double combination of MOX and RPT, as used in the standard Denver regimen against TB [25], is highly potent in depleting *Wolbachia* in the *L*. *sigmodontis* larval model.

## Discussion

Innovative treatments are urgently needed to improve individual clinical care for patients, reduce human suffering, and ensure advancement towards the WHO and ESPEN (Extended Special Project For Elimination Of Neglected Tropical Diseases) goals for LF and onchocerciasis elimination (http://apps.who.int/gb/ebwha/pdf_files/WHA70/A70_35-en.pdf). Depletion of *Wolbachia* with DOX has resulted in the development of an alternative anti-filarial chemotherapy, with a 4–6 week treatment course resulting in long-term sterility and most importantly a strong macrofilaricidal effect (even though temporally delayed) [15, 27]. The indirect mode of action results in a slow death of the adult worms and this “soft kill” is a highly desired outcome in order to avoid the rapid release of parasite and *Wolbachia* products that can induce a strong pro-inflammatory response and thereby adverse side effects [17]. The A-WOL consortium has focused on the discovery and development of new or repurposed antibiotics without the caveats of DOX (long-term treatment, not approved for use in children and pregnant women) [4].

Here we describe the use of the rodent *L*. *sigmodontis* model as an *in vivo* screening system for the discovery and development of anti-wolbachial compounds. This screening procedure that examines anti-wolbachial drug efficacy during L3 to adult worm development is a very sensitive tool to assess anti-wolbachial drug candidates. It has the advantage to identify also non-optimal candidates that need further treatment or formulation adjustments rather than missing potential hits. At this stage parasite replication occurs at a relatively high level and we could show that bacterial function is acutely needed for the further larval development and growth. Using this sensitive stage therefore allows for the identification also of non-optimal candidates that may only need further dose or formulation adjustments, which would be missed otherwise. However one must be cautious as to whether treatment of the larvae is a good predictor for the treatment of adult parasites in humans. It may well be that dose and duration of treatment may be higher and/or longer for an anti-wolbachial drug to deplete *Wolbachia* also in adult worms. Therefore within the development of a hit/lead into a preclinical candidate, studies must include those that treat animals after the onset of patency. This requires the use of jirds, which without treatment stay patent for at least 6 months.

Also, modelling has to be applied involving PK/PD data from different animals such as mice and jirds (and for PK, larger animals such as dogs) in order to predict appropriate bioavailability and efficacy in humans. Such models have been developed [28]. When starting the treatment (DOX, 50 mg/kg/14d) one day after infection, *Wolbachia* numbers remain suppressed in the treatment group until the last follow-up in the larval screen at day 35, the time point when larvae have otherwise passed their final moult into adult worms. In contrast, a larval stage (L3-L4, L4-L5) and worm size dependent increase of *Wolbachia* occurred in the control group (Fig 1b, [29]). Absence of *Wolbachia* was not only associated with the inhibition of embryogenesis [8, 10] and obvious growth retardation (Fig 1c, [10, 30], but also a developmental arrest at the L4-stage (Fig 2e), an observation that was noticed earlier in *Brugia pahangi* infected gerbils [31]. This finding suggests a fundamental role for *Wolbachia* not only for reproduction and survival of the parasites but also for growth and development. In addition to the biological importance itself, this finding adds another benefit to this model. Terminating the experiment at a time point where control worms have moulted into adults allows a quick evaluation of efficacy of a given compound by assessing growth retardation. This is supported by our data using suboptimal concentrations of antibiotics suggesting that growth inhibition precedes absence of *Wolbachia*, indicating functional inhibition of bacteria-derived molecules required to enable parasite development. This is particularly important in order not to prematurely reject drugs administered at suboptimal dose during screening, or hits that need further hit to lead optimization, or drugs that show bacteriostatic rather than bactericidal effects. In the latter, biological effects of partial *Wolbachia* depletion like growth inhibition are present, although *Wolbachia* are still detected by PCR (Fig 4a–4c). The concept of functional rather than numerical impairment of *Wolbachia* is of biological importance, but difficult to be directly translated into the therapy of human infections, as a different stage is targeted, i.e. the adult worm. The model focussed upon in this study targets the larval stage, a phase in which bacterial replication occurs at very high levels and a potential repopulation of *Wolbachia* may occur. In human clinical trials however it could be shown that a greater than 90% reduction of *Wolbachia* does not lead to reoccurrence of bacteria, a threshold that is needed for successful treatment efficacy.

To establish this model for different routes of administration, we compared the ip and oral administration routes and performed dose titration experiments. After both, oral and ip application of DOX, a dose dependency occurred with regard to bacteria depletion and inhibition of filarial growth (Figs 2 and 3). Despite the fact that the oral application is less effective than the ip administration due to DOX’s known poor oral bioavailability [32], oral or ip application of a compound can be tested in this model, depending on the requirements of the research question and the pharmacokinetic profile of the compound. We further suggest that for the identification of compounds with better potency than DOX, a DOX regimen reduced in both dose and time should be used (25 mg/kg/10d) and compared to the gold standard (50mg/kg/14d). An example for such a drug is MIN, another broad-spectrum tetracycline antibiotic that has already been found to be more active than DOX *in vitro* using *Onchocerca gutturosa* adult males [33]. MIN was among the top hits of the A-WOL screening activities [4] and its efficacy was recently confirmed using the *Wolbachia* containing insect cell line C6/36 [19] and adult *Brugia malayi* screens [34] and in a human clinical trial [35].

Using the suboptimal regimen (25mg/kg/d/14d), DOX showed intermediate levels of *Wolbachia* reduction, whereas the biological activity of the bacteria was already blocked, as seen by filarial growth inhibition. In contrast, tigecycline and MIN were even more potent in *Wolbachia* reduction compared to DOX. Whereas the application of tigecycline is intravenous and therefore does not conform to the current TPPs for macrofilaricidal drugs, MIN is a potential candidate, although it has, as a tetracycline, the same contraindications as DOX [34]. Rifamycins may overcome those contraindications and our study showed that ip treatments with RIF and RPT alone for one week reduced the *Wolbachia* burden by 97 and 99%. Recent PK/PD analysis and *in vivo B*. *malayi* and *O*. *ochengi* studies suggest that orally administered RIF at an elevated dose of 35 mg/kg leads to a *Wolbachia* reduction greater than 90% predicting cure in *B*. *malayi* and *O*. *ochengi* after 7 and 14 days of treatment, respectively [28]. Due to this increased *in vivo* potency elevated RIF dose treatment is thought to achieve equivalent efficacy as the long-course DOX treatment and is proposed for phase II trials.

For some important infectious diseases, combination therapies with antibiotics have been established. An important example is the standard Denver regimen for treatment of tuberculosis, consisting of a 6-months course of a combination of isoniazid and RIF administered daily, supplemented by ethambutol and pyrazinamide in the first two months. Approaches to shorten the treatment time or reduce the number of daily observed treatments is an important goal that will increase adherence to the treatment and thereby treatment success. Investigations have compared daily RIF versus RPT and have shown that once weekly RPT and daily RIF have the same efficacy with similar cure rates [36], however, with an increase of the risk of bacteriological relapse [37]. Further studies focused on the increase of the dose of RPT from 10 to 15 mg/kg to compensate for its high protein binding that may be partially responsible for the suboptimal activity observed in once-weekly regimens [38]. The 15 mg/kg dose of RPT has been well tolerated in humans and increasing the dose of RPT has demonstrated enhanced sterilizing activity in a mouse model of tuberculosis [39]. In our studies, we showed that RPT was superior to RIF depletion with regard to *Wolbachia* depletion in the *L*. *sigmodontis* model. Additional improvement was achieved by the inclusion of MOX, a fluorquinolone of the fourth generation with potent bactericidal activity [25]. This regimen has been investigated recently in a clinical trial in humans for TB and it has been shown that the 6-month regimen with a weekly administration of high-dose RPT and MOX is as active as the control regimen and the 4 month regimen is non-inferior to the control regimen [40]. However this treatment is not recommended for general use other than progressing active TB in children and pregnant women.

We tested in our model whether a combination of a tetracycline, rifamycin and MOX were able to reduce the treatment duration and found high efficacy in the reduction of bacterial loads with RPT and MIN being slightly more potent than RIF and DOX.

Testing all double combinations that could be built from the triple combinations revealed that the combination of RPT (15mg/kg) and MOX (2 x 200mg/kg) had the best efficacy in both administration routes (oral or ip). The RPT plus MOX combination was equivalent to the triple combination (>99% *Wolbachia* reduction).

These investigations suggest that it may be possible to shorten anti-wolbachial treatment times to 7 days or less in humans, meeting the criteria of the current TPP for macrofilaricidal drugs. In addition, those shortened treatment times have the advantage that they are unlikely to cause resistance in *Mycobacterium tuberculosis*. Further PK/PD studies similar to Sharma et al. [34] and screening in other models of adult filarial infection [41] should be performed to confirm pan-filarial activity and to better identify the human bioequivalent doses to translate these results successfully to clinical trials.

## Supporting information

S1 Table Log drop and significances of all experiments. (XLSX)
